# Therapists’ experiences with providing guided internet-delivered cognitive behavioral therapy for patients with mild and moderate depression: a thematic analysis

**DOI:** 10.3389/fpsyg.2023.1236895

**Published:** 2023-07-14

**Authors:** Line Børtveit, Tine Nordgreen, Anders Nordahl-Hansen

**Affiliations:** ^1^Faculty of Health, Welfare and Organisation, Østfold University College, Halden, Norway; ^2^Department of Behavioral Sciences, Faculty of Health Sciences, Oslo Metropolitan University, Oslo, Norway; ^3^Division of Psychiatry, Haukeland University Hospital, Bergen, Norway; ^4^Department of Global Public Health and Primary Care, University of Bergen, Bergen, Norway; ^5^Department of Education, ICT, and Learning, Østfold University College, Halden, Norway

**Keywords:** internet-delivered therapy, therapists’ experiences, providing online therapy, iCBT, internet-delivered therapy for depression, thematic analysis

## Abstract

**Introduction:**

Guided internet-delivered therapy has shown promising results for patients with mild and moderate depressive disorder, but several challenges with the format have been reported. The aim of this qualitative study was to investigate therapists’ experiences providing guided internet-delivered cognitive behavioral therapy for patients with mild and moderate depression.

**Material and methods:**

Twelve therapists were interviewed, and the interviews were analyzed using reflexive thematic analysis.

**Results and conclusion:**

Three themes were created: (1) *For the right person, at the right time*. This theme is about therapists’ experiences appointing patients to the program. It is challenging to predict which patients will benefit from it, and it is not the right option for all patients. (2) *It is not like chatting on Facebook*. The second theme was about the experiences with demands on clinics, therapists and patients that must be considered. The internet-delivered treatment should not be viewed as a simple treatment option, and the value of having contact with the patients during treatment was emphasized. (3) *It is like a railroad, but without the switches*. This theme was about the experiences with how the treatment content was conveyed to the patients, how the therapists expressed concerns with the usability of the program and the reported need for more possibilities in tailoring treatment for each patient.

## 1. Introduction

Depressive disorders have been among the top three leading causes of non-fatal health loss and disability for nearly three decades, affecting more than 264 million people worldwide (James et al., 2018). The disorder is associated with distress, increased risk of death by suicide (Bostwick and Pankratz, 2000) and high societal costs (Greenberg et al., 2003; Stewart et al., 2003; Sobocki et al., 2006; Kessler, 2012). Access to effective treatment is a challenge (Wang et al., 2007; Moitra et al., 2022) and few patients receive adequate treatment (Kessler et al., 2003; Kazdin, 2017; Thornicroft et al., 2017). Developing effective and accessible psychological treatments for depressive disorders presents a global challenge as mental health problems are an increasing global burden (Carey et al., 2017).

### 1.1. Internet-delivered therapy

Meta-studies have shown that therapist guided internet-delivered interventions are effective in treatment for mild and moderate depressive disorders, and few patients (5%–10%) experience deterioration or negative effects (Cuijpers et al., 2015; Rozental et al., 2015; Ebert et al., 2016; Karyotaki et al., 2018; Etzelmueller et al., 2020; Chan et al., 2022; Hedman-Lagerlöf et al., 2023).

Typically, in internet-delivered therapies, the treatment content is delivered to the patients via apps or webpages and the patient works with different modules composed of reading assignments and tasks (Schröder et al., 2016). These treatments could include guidance and support from a therapist, but there are also many examples of unguided treatments (Berger et al., 2011; Krämer et al., 2022). For internet-delivered interventions directed at patients with depression the treatments are often based on approaches frequently used in treatment of this patient group, such as *cognitive behavioral therapy* (CBT), problem solving therapy, behavioral activation, mindfullness and acceptance and commitment therapy (Brown et al., 2016a; Karyotaki et al., 2018; Garrido et al., 2019; Etzelmueller et al., 2020; Børtveit et al., 2022).

### 1.2. Therapists experiences

Common reported challenges are related to usability, interactivity and treatment dropout (Van Ballegooijen et al., 2014; Nordgreen et al., 2019). Low intake, and skepticism among general practitioners and therapists to the treatment format is also reported (Folker et al., 2018). Therapist experiences are therefore of importance and have shown to be valuable when implementing, evaluating and designing digital mental health interventions (Brown et al., 2016a,b; Dekker and Williams, 2017; Hadjistavropoulos et al., 2017; Kadesjö Banck and Bernhardsson, 2020; Stawarz et al., 2020; Freund et al., 2023; Tarp et al., 2023).

Previous studies focusing on the therapist’s experiences with therapy designed to be delivered online, points to generally positive attitudes to the treatment format (Kerst et al., 2020; Wood et al., 2021; Khan et al., 2022; Vis et al., 2022). Positive attitudes among the therapists were also reported in studies where internet-delivered therapy is provided in combination with face-to-face sessions (Titzler et al., 2018; Mol et al., 2020).

#### 1.2.1. Need for training and supervision

One commonly reported challenge therapists encounter when implementing internet-delivered treatment is that there is a need for sufficient training and supervision (Folker et al., 2018; Meisel et al., 2018; Titzler et al., 2018; Kerst et al., 2020; Smith and Gillon, 2021; Wood et al., 2021; Doukani et al., 2022; Vis et al., 2022). The training should be specific to the platform that is used in the intervention (e.g., how to navigate the webpage or app) (Wood et al., 2021), and directed at developing and altering therapists’ skills and approaches to that of an online format (e.g., how to build a therapeutic alliance without face-to-face meetings) (Smith and Gillon, 2021). To have a dedicated implementation team, that arranges group training where sharing experiences is encouraged and provides ongoing supervision, is described as an important facilitator (Meisel et al., 2018; Wood et al., 2021; Vis et al., 2022).

#### 1.2.2. The therapeutic relationship

The therapeutic relationship or alliance between therapists and patients could be affected by the online format (Kotera et al., 2021). Meisel et al. (2018) concludes that the perceived lack of a therapeutic relationship is a barrier that could hinder therapists from offering internet-delivered treatments. Titzler et al. (2018) found that limited face-to-face sessions and technical issues in their interventions affected the establishment of therapeutic alliances between patients and therapists. Doukani et al. (2022) describes that these problems could be addressed by making sure that the therapists have enough time to deliver the treatment and do not experience time pressure as well as increase opportunities to tailor the treatment to the patient’s needs and provide appropriate training for the therapists. Other studies have shown that therapists were able to effectively develop therapeutic alliances with patients in an online format (Bengtsson et al., 2015; Borghouts et al., 2021; Wood et al., 2021; Khan et al., 2022). Wood et al. (2021) and Bengtsson et al. (2015) described that some therapists experienced that therapeutic relationships could be developed effectively, and in some cases even faster compared to traditional therapy because the patients felt more comfortable to disclose more rapidly. Therapists that participated in the study by Khan et al. (2022) described that they felt able to stay present and attuned to the patients’ emotional states, and could thereby build confiding relationships with those patients. In Borghouts et al. (2021) social connectedness, developed during online treatment, was described as something that was facilitated by the intervention. Smith and Gillon (2021) emphasizes the need for sufficient training of therapists to secure emotional connectedness and therapeutic alliance in internet-delivered treatment programs.

#### 1.2.3. Personalization and tailoring of the treatment

Lack of personalization or possibilities to tailor the internet-delivered treatment is described as a key barrier for implementation (Titzler et al., 2018; Borghouts et al., 2021), and is presented as an important factor to consider in future intervention development. The possibilities to tailor the treatment to each patient is in several studies described as important (Bengtsson et al., 2015; Folker et al., 2018; Titzler et al., 2018; Borghouts et al., 2021; Doukani et al., 2022). In most traditional face-to-face therapy sessions, the therapists generally are free to make individual adaptations. In many internet-delivered treatment programs this freedom is more restricted. The intervention might follow a predetermined plan, where each session focus, assignments and tasks are fixed, and few adaptations to accommodate individual needs can be made. At the same time, to have a pre-determined structure of the treatment could be experienced as beneficial. Bengtsson et al. (2015) described that the manual-based internet-delivered therapy made it easier to achieve focus compared to face-to-face sessions, where there were more possibilities of going off on tangents or getting stuck on a topic. Mol et al. (2020) found that the pre-set structure of their interventions made both therapists and patients adhere to the treatment protocol, and reduced therapist drift. But in both of these studies the lack of flexibility was described as a challenge, especially for less experienced therapists who reported that the protocol felt too structured and inflexible. The therapists in these studies described that they often made some adaptations and additions to the manualized program. This might be easier in a blended intervention where internet-delivered treatments are combined with some face-to-face sessions.

#### 1.2.4. Technical support

Technical issues have also been central as a barrier for internet-delivered treatment in several recent studies (Titzler et al., 2018; Mol et al., 2020; Borghouts et al., 2021). Therapists expressed concerns with being unable to assist patients with technical issues and some experienced lack of support from the helpdesk (Mol et al., 2020). To have access to necessary resources such as technical support was described as important, and a characteristic of a supportive organizational climate for implementation of internet-delivered treatment for depression (Vis et al., 2022). Khan et al. (2022) concludes that technical difficulties did not hinder the therapists in building good relationships with their patients, which was a concern presented by Titzler et al. (2018).

#### 1.2.5. Personal effort, motivation and responsibility for treatment

Internet-delivered interventions typically require that the patients work independently and put high personal effort into their own treatment. In therapists-guided interventions the role of therapists is to provide support to their patients, who work with the self-help material (Schröder et al., 2016). The patient’s interests, willingness and motivation to adhere to the treatment is therefore experienced as an important factor and described as a key facilitator for treatment (Bengtsson et al., 2015; Titzler et al., 2018). The online treatment format gives the patients a feeling of more autonomy compared to what would typically be the case during face-to-face therapy (Wood et al., 2021). A potential challenge, despite the high amount of independent effort made by patients, is for therapists, who provide internet-delivered therapy, to accept the boundaries the online format implies and deal with the sense of less control and vulnerability in how they manage aspects of risk (e.g., risk of deterioration, suicide or self-harm) (Smith and Gillon, 2021; Wood et al., 2021). One advantage, to giving patients the responsibility for their own treatment, is that the patients after completion of the treatment would have gained new skills and be more empowered to handle future depressive episodes (Lillevoll et al., 2013; Berg et al., 2020; Gericke et al., 2021; Lindegaard et al., 2021; Eilert et al., 2022). Therapists in Titzler et al. (2018) described that the patients kept access to the online content even after the therapy was ended, which was experienced as a positive aspect of online intervention.

### 1.3. Purpose and aim

Internet-delivered interventions could be an effective and available treatment option for patients with depression, but several challenges have been reported, e.g., in relation to treatment drop out and adherence (Van Ballegooijen et al., 2014; Børtveit et al., 2022), limited possibilities to tailor treatment to patient’s needs (Titzler et al., 2018; Borghouts et al., 2021) and how the format affects the patient-therapist relationship (Kotera et al., 2021). To provide better interventions research investigating and illuminating challenges experienced in these interventions are called for.

The purpose of this study is to investigate therapists’ experiences providing guided iCBT to patients with mild and moderate depressive disorder.

## 2. Materials and methods

### 2.1. The eCoping program

The eCoping program is a *therapist guided* iCBT intervention for patients with mild and moderate depression. The eCoping program is based on an intervention designed by Andersson et al. (2005) and adapted to Norwegian users. The program is module based and consists of eight modules, all modules and a short description of their content is presented in Table 1. The program is completed over approximately 14 weeks. Asynchrony contact with feedback and guidance is provided in an integrated secure email-system after completion of modules, but more contact (e.g., over phone) is provided as needed. The program has effectively been used in outpatient clinics in Norway (Jakobsen et al., 2017; Nordgreen et al., 2019).

**Table 1:** Modules and content in the eCoping program for depression.

Module	Topic	Content
1–2	Psychoeducation: CBT and depression	CBT approach, depression, behavioral activation, etiology
3–4	Psychoeducation: activities	Positive and negative activities, short- and long-term effects, choosing activities, avoidance, punishers, problem-solving, rewards
5	Psychoeducation: depressive thinking and rumination	Negative automatic thoughts, interpretations, techniques for shifting focus and being mindful, problem-solving
6	Negative thoughts	Approaches to defuse negative thoughts. Acceptance and shifts in focus towards valued activities
7	Sleep and relaxation	Sleep hygiene and relaxation techniques
8	Summary. Setbacks and relapses	Summary of all modules. Preparing for setbacks and preventing relapse.

CBT, cognitive behavioral therapy.

### 2.2. Procedure and participants

In order to gather in depth information about therapists’ experiences with the eCoping program, we conducted individual qualitative interviews with 12 experienced therapists (Table 2). Eligible participants were therapists with experience using the iCBT program for patients with depression. The therapist should at least have experience from treating one patient in the program.

**Table 2:** Participating therapists.

Pseudonym	Profession	Number of patients in iCBT for depression
Anne	Psychologist	10–20
Camilla	Psychologist	1–5
Daniel	Occupational therapist	>20
Thor	Social worker	1–5
Katrine	Psychologist	1–5
Kristoffer	Psychologist	>20
Maia	Psychologist	1–5
Marianne	Nurse	1–5
Monika	Psychologist	1–5
Sarah	Psychologist	6–10
Sofie	Psychologist	6–10
Thomas	Psychologist	6–10

We recruited therapists from December 2020 to March 2022. An invitation for participation in the study were sent to several psychiatric outpatient clinic in Norway where the eCoping program was offered to patients with depression. Due to restrictions following the COVID-19 pandemic most of the recruitment was conducted over e-mail.

We developed and used an interview guide to prompt the interviewer, and a semi-structured interview process was followed. The initial questions were general questions about the experiences with the program, and interviewees were prompted to speak freely about the topics they considered most important. More specific questions were asked later, or as follow-up questions in line with recommendations from Braun and Clarke (2013). Topics covered in the interviews where about the intervention (e.g., how did you experience this treatment program?), facilitation (e.g., were arrangements made for this way of working at your workplace?), usability (e.g., how was it to navigate the webpage?), acceptability (e.g., do you think this treatment is full-fledged or is there something you are missing?) and satisfaction with the program (e.g., would you recommend this kind of treatment to friends or acquaintances with depression?) The whole interview guide is provided in Supplementary material, Appendix A. We reviewed the interview guide at several points during data collection, and as recommended by Braun and Clarke (2013) made changes to embrace topics we discovered during the interviews.

Two interviews were carried out in the therapist’s offices (with participants Sarah and Kristoffer). Due to restrictions following the COVID-19 pandemic this was not possible for the remaining interviews, and they were conducted using a video conferencing tool (Whereby). The audio was recorded and after the interviews LB transcribed the interviews, inserted pseudonyms and removed identifying information. All participants received a 300 NOK (equivalent to 30 USD/25 EUR) gift-card as thanks for giving their time to the project.

Ethical approval was granted by the Norwegian Regional committees for medical and health research ethics, case number: 171,864. All participants signed a consent form before participation.

### 2.3. Analytic approach

Aiming to capture and explore the participating therapists’ experiences we approached the analysis with an experiential qualitative approach (Braun and Clarke, 2021) and *reflexive thematic analysis* (TA) (Braun and Clarke, 2006, 2013, 2019, 2021) was selected as the most appropriate method to analyse our data. This method seemed suitable as the user’s experiences was at the center of our research, at the same time it enabled us to focus on “the bigger picture.” We believed that this method would provide a suitable framework for interpreting the data, creating results that would be pertinent in developing future interventions, and providing an overview over the possible challenges and facilitators the therapists’ encounters. We leaned towards a realist/essentialist approach where the aim of the analysis was to “*capture truth and reality, as it is expressed within the dataset*” (Braun and Clarke, 2021, p. 10). The reflexivity of reflexive TA makes it possible to summarize and interpret the qualitative data in the context of the experiences and realities of therapists as well as the context of us as researchers.

### 2.4. Researcher positioning

Given the researchers’ active roles in reflexive TA, the context or positioning of the researchers should be described (Braun and Clarke, 2006, 2023). LB is a researcher working on a project focusing on internet-delivered treatment for depression. She has no former experience with providing therapy or working with patients who are diagnosed with depression. When conducting the interviews with the therapists, she informed about her “outsider” perspective. TN has been working with the development, evaluation and implementation of guided iCBT for the last 15 years. AN-H is a researcher in the transitional fields of special education, psychology and psychiatry, and have clinical experience working in the psychiatric field for 12 years.

### 2.5. Coding and creation of themes

Following the six phases in reflexive TA presented by Braun and Clarke (2006) we started by reviewing the transcripts and made notes of initial codes. In the coding process we had an inductive approach. We based codes on the surface meanings, and viewed what the participants told as a reflection of their experiences and reality, hence the analysis had a semantic approach (Braun and Clarke, 2021, pp. 9–10).

#### 2.5.1. Coding

The coding was conducted over several sessions. Parallel to the coding interesting topics and ideas for potential themes were noted in a separate document. LB conducted all coding, which started with the creation of an extensive list of codes using direct words, sentences and summaries of statements from the transcripts in a Microsoft Word document. The codes and transcripts were reviewed and imported to NVIVO (QSR International Pty Ltd, 2020), and codes were merged to represent a broader content so that the number of codes were reduced. Sub-codes were also used. At the end of this stage 66 codes and sub-codes had been developed. The 66 codes were then reviewed by using sticky notes that made it possible to organize and reorganize over several sessions to create a starting point for creation of themes. Three potential themes were created in this stage. To secure easy access to all codes and corresponding quotations we recreated the structure from the sticky notes in NVIVO.

#### 2.5.2. Creation of themes

The potential themes were defined, with descriptions of what they would contain, what they would not contain, and how the themes were linked together. TN and AN-H evaluated the potential themes and provided feedback to LB.

The themes where then evaluated in relation to the codes. This resulted in some changes in the third theme that we changed from being a summary of the interventions usability to a theme capturing the therapists’ experiences using the intervention to deliver treatment content. For the second theme this process highlighted the need for sub-themes to organize the content better.

## 3. Results

**Figure 1:**
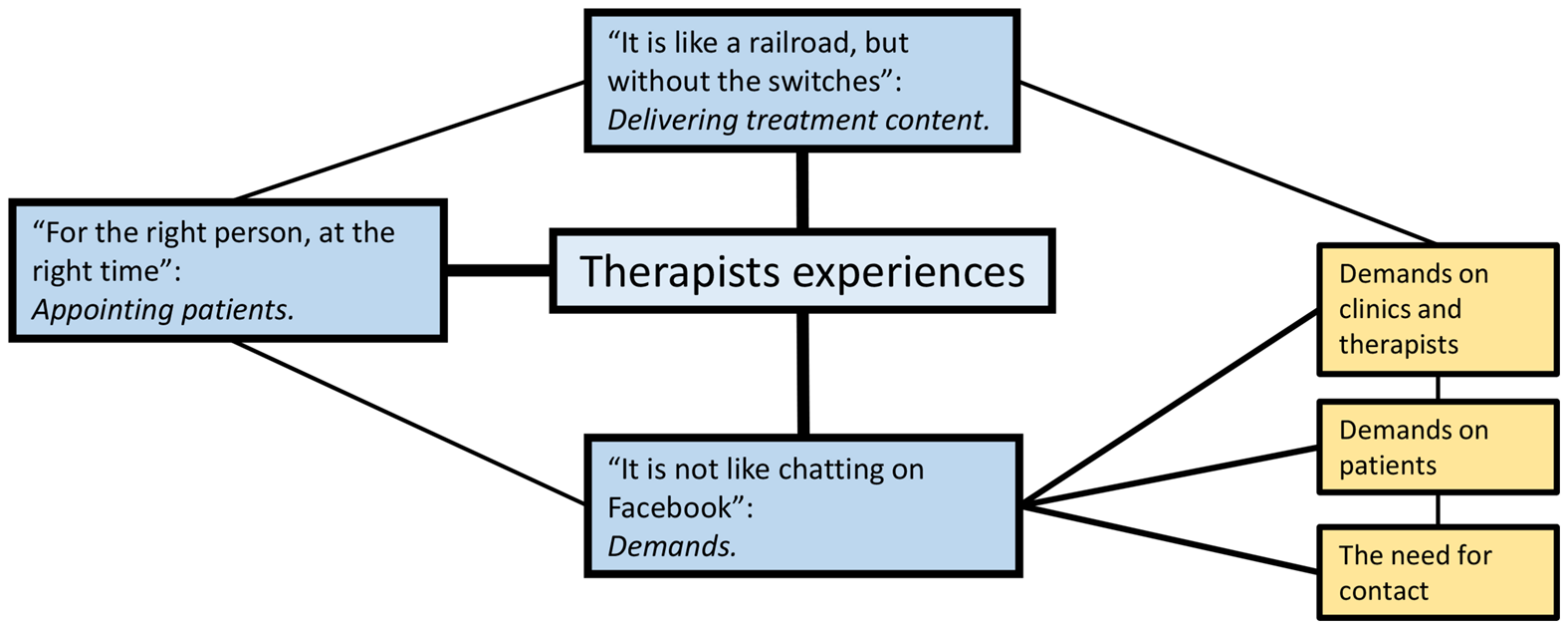
The three themes, and subthemes created to reflect the therapist’s experiences with providing guided internet-delivered therapy for patients with depression.

We created three themes, and sub-themes (see Figure 1), a detailed description of the themes is presented in Supplementary material, Appendix B. Theme 1—“*For the right person, at the right time.”* This theme is about the therapists’ experiences with appointing patients to the guided iCBT program. Theme 2—“*It is not like chatting on Facebook.”* This theme is about the demands that need to be considered with this type of treatment. Three sub-themes were created for this theme: *Sub-theme 2A—The demands on the clinics and therapists, Sub-theme 2B—The demands on the patients, Sub-theme 2C—The need for contact between therapists and patients*. Theme 3—“*It is like a railroad, but without the switches.”* This theme is about how the therapists experience delivering treatment content using the guided iCBT program.

### 3.1. Theme 1: for the right person at the right time

The first theme we created captures the challenges that therapists reported when assigning patients to the guided internet-delivered treatment. There was a consensus among the therapists that even though guided iCBT treatment could be effective and useful, it was not the right treatment option for every patient. Furthermore, it should only be selected for “*the right person, at the right time”* (Thomas). The therapists expressed it as important and challenging to assess which patients would benefit from the program:

The biggest challenge is to select patients to the program. Who is it fitting for? I find it difficult, (…) sometimes there are patients where I (…), I have so little faith in it, but then it goes well, and other times where I have thought, well, well, here it would fit very well, and then it does not work. So that is a little challenging. (Camilla)

There was a consensus among the therapists that before the patients were appointed to the guided iCBT, a thorough assessment had to be conducted. However, it was unclear for the therapists what they should primarily look for during this assessment. Prerequisites for successful treatment were described as sufficient language skills, reading comprehension, eye sight and computer skills. Another important factor, frequently reported as a challenge to assess, was patients’ motivation. The patient’s willingness to actively be involved in the treatment was emphasized by several therapists: “…*they must be ready for action…”* (Thomas). The patient’s motivation and willingness to partake in the treatment was a challenge for therapists to predict beforehand, and could even change, both positively and negatively, during treatment.

Patients that dropped out from the treatment was described as something therapists experienced as demotivating:

What I recognize is a feeling of demotivation as a therapist when you are unable to select well and you have several patients who do not succeed at all. (Marianne)

Dropping out of treatment could have negative effects on the patient, the therapist, and the general perception of the treatment program. Thus, therapists strived to only include those patients with necessary prerequisites to complete treatment. In addition, therapists advocated for a flexible approach if internet-delivered treatments should be discontinued due to a lack of treatment effect.

The ideal patient to appoint to guided iCBT program was described as someone that had the ability to prioritize his/her time towards the treatment and had few to no other personal complications beyond the diagnosis of depression to contend with simultaneously. Furthermore, activities such as work, and support from family and friends could be an important factor for treatment success. The therapists reported that the need for patients to carry out the treatment with a high degree of independence indicated that these patients must be disciplined, conscientious and have room for the treatment in their everyday life:

You need to have the opportunity in your life, she (one patient) was in a very challenging phase with young children, problems, lot of work, many things… so it, it simply became to challenging. (Sofie)

Due to the need for high patient involvement and effort, this problem was explained as perhaps even more pressing when using the internet-delivered treatment format compared to face-to-face therapy. It was likewise emphasized that this type of treatment should be chosen by the patients. The therapists reported that if guided iCBT was presented as the patients only treatment option this could have negative effects on their motivation and willingness to put the needed effort into the program. Reduced resources and availability of therapists in the clinics should not be used as justification for appointing patients to the online treatment. There has to be a genuine belief that this program would be a good option to help the patient feel better.

the pitfall is that it is presented as a money-saving method… because you in theory could have more patients in treatment (…) alternatives should be presented (to the patients), it should not be like you could get treatment, but only eCoping, that’s our only option. I have little faith in that… (the treatment should be) …an active choice (…) it is so demanding (for the patients), that if this is not the case we will end up with a lot of dropouts…. (Katrine)

The therapists further explained that due to the symptoms with depression, these symptoms can diminish further the success with the treatment and in particular with motivation, initiative and willingness for action:

…a common effect of depression is difficulties with getting things like this done, and I think it is kind of ironic that what you try to treat, at the same time is something that makes the needed actions difficult to do. (Kristoffer)

This would also influence which patients that should be appointed to the treatment. Some of the therapists experienced that patients receiving treatment through a specialist health care service, are so ill that the online-format would not be appropriate for most of them. Many of these patients might need closer monitoring and more attention from a therapist.

### 3.2. Theme 2: it is not like chatting on Facebook

The therapists reported that to appoint patients to the guided iCBT program should not be viewed as a simple or easy solution for either the therapists or the patients. The therapists described several demands that need to be considered. For the second theme, we created three sub-themes which cover these demands:

#### 3.2.1. Sub-theme 2A: the demands on the clinics and therapists

The therapists expressed that the internet-delivered treatment format provided flexibility and a welcomed variation in their workdays, but the format also puts high demands on them. The therapists reported that the guided iCBT required that they put in a lot of effort, time and devotion to their patients:

…it’s this about time use, it has to be appointed enough time for it, it has to be acknowledged that this is treatment, it is not a simple solution. I think the focus is on that this is a full-fledged treatment for the patients, without considerations about the effort it takes for the therapists (…) it demands quite a lot (…) it is not like chatting on Facebook. (Marianne)

Several therapists felt that it was challenging to keep the allotted time devoted to the iCBT patients, the time was often filled with other patients, meetings and other tasks. Different strategies to keep their calendar clear of other appointments was presented (e.g., working from home), and more accommodation at the clinics was described as necessary.

The importance of sufficient training and supervision provided by the clinics was communicated in the interviews. The therapists also expressed that the clinics should facilitate for collegial community at the workplace, with several therapists in close proximity (e.g., sharing an office) working with the program. This made it possible for the therapists to help each other with technical issues and provide feedback and input on patient cases. A collegial community at the clinic served also as a well-being aspect for the therapists:

It is nice that we are a team, the possibility to discuss cases with others who also work like this, I think that is… central to it, that it is fun to do this…. (Camilla)

The therapists reported that it was important that clinics facilitated with the possibility of combining online therapy with other tasks. None of the therapists asked would have worked full time with guided iCBT. The variation seemed to be important and provided what several therapists described as a break from the traditional face-to-face sessions:

I like it (…) it gives me some variation in the everyday work routine. (Maia)

#### 3.2.2. Sub-theme 2B: the demands on the patients

The guided iCBT treatment was viewed by the therapists as a treatment that places high demands on the patients to take responsibility for their own treatment.

…it does take time. We have to be honest about that, it takes time to complete this treatment, it is what it is, it is treatment. And it demands high personal effort. (Marianne)

To place a large amount of the responsibility on the patients for the treatment was experienced as something that made the treatment more available and increased flexibility as patients were free to work with the treatment whenever they had the opportunity. More flexibility makes the treatment more accessible for those patients who might find it challenging to adhere to traditional therapy (e.g., working shifts making it challenging to go to the therapist’s office at day time). But the flexibility also demands that these patients set aside time and prioritize the treatment. The therapists expressed that this could be difficult for many patients due to the symptoms of depression including the difficulties with concentrating, focusing and taking initiative as well as setting time aside for treatment when their lives are quite busy already.

…It could be challenging to make it (the treatment) work due to the everyday life, and it could be challenging to make it work due to the condition (…) in regards to how they are cooping, the lack of initiative and concentration… energy. And determination…. (Katrine)

Some of the therapists raised concerns that the flexibility of the treatment could lead to procrastination and avoidance: “*eCoping therapy is a golden opportunity for avoidance*” (Sarah), and elaborated that the patient’s responsibility should increase gradually during treatment. Others attributed lack of success to the inclusion process, where patients that are not able to handle the demands the treatment format and thus should be given other treatment options.

Positive effects of placing responsibility for treatment on the patient were also discussed:

To become your own therapist, it is the goal, building therapy, they should have learned something they could take with them, and I think therefore that there are some great advantages in these high demands for personal effort. (Camilla)

#### 3.2.3. Sub-theme 2C: the need for contact

To build a therapeutic alliance the therapists emphasized the importance and value of having contact with the patients during treatment. Contact with the therapists functioned as a channel for help, encouragement and motivation for the patients during treatment. Some patients choose to have limited contact with their therapist during treatment, but the therapists still considered the possibility to have contact as an important part of the program. With a large part of the guided iCBT requiring patients to work independently on the program, emailing with the therapist might provide additional support and comfort for the patients:

(the patient) is not completely alone…. (Monika)

The possibility to have contact in other ways (e.g., phone calls, video conferences or physical meetings) was experienced as important by the majority of the therapists. Patients in need of more contact were encouraged to contact the therapists. The possibilities to offer other contacts, besides the email system, should be considered when therapist guided internet-delivered therapy is provided. At the same time, the need for contact is something that should be evaluated when patients are appointed to the treatment:

For some patients there is a need to come here and talk to someone. We should not forget that. Things like that. The program does not fit for everyone. (Thor)

The therapists revealed that they felt that each message they wrote to the patients was of high importance due to the limited contact between them. The interviewed therapists reported that they seldom used the pre-written messages in the program, and they used time and effort to provide well-written messages. The emailing and other forms of contact with the patients were described as a way for therapists to tailor the treatment for each individual patient in the program. But it was also viewed as a difficult challenge to balance the helpful and motivating messages so that they were not experienced as discouraging to the patients.

### 3.3. Theme 3: it is like a railroad, but without the switches

The third theme was created to capture the experiences the therapists had when using the guided iCBT program to deliver the treatment content to their patients. Most therapists approved of the treatment content, and found it useful and relevant. They encountered few technical difficulties and reported that they felt that data security and protection was handled well. The therapists experienced that the secure login process made the patients feel that they entered a closed room, where they felt safe to share, like a therapy room.

The treatment content was divided into several smaller modules and was viewed as advantageous as it made the amount of content the patients got access to more manageable. The therapists also expressed it as positive that the guided iCBT program secured the patients’ ability to get access to the treatment content in a predetermined and planned way:

I think that it (eCoping) is a good treatment, it might even be better on certain areas than other treatment we offer (…) its content is controlled and we know what it is, as opposed to other types of treatment where we know that it could vary a lot what kind of treatment you actually end up with. (Kristoffer)

But the predetermined way the treatment was delivered was also reported to make the treatment feel inflexible with limited possibilities in tailoring the treatment for each patient. Several therapists declared this as highly problematic where patients had to work their way through several of the programs modules to get to the ones that addressed the patients most pressing symptoms or problems. The therapists described that this potentially could lead to dropout, or lack of effect in earlier modules:

it is like a railroad, but without the switches, so if you choose the wrong program from the start, you are stuck here, or we have to terminate the treatment. (Daniel)

To have the possibility to change the order of the modules to tailor the treatment for their patients was something the therapists missed in the program. Several therapists shared that they compensated for the lack of flexibility by asking their patients to skip, or take it easy on the modules that they felt were not relevant. But with modules that are consecutively built upon each other, this was also viewed as problematic. The lack of possibilities to tailor the treatment for each patient made some of the therapists express that the program felt a bit like a “one size fits all” solution, where the severity of the depression was trivialized. Other told that patients in need for more tailored treatments should be appointed to other treatment options.

The therapists emphasized the need for the treatment to be as usable and accessible as possible but did point to the challenge of delivering a large amount of treatment content in textual form:

It is a challenge when you are down, feeling down, depressed, lack concentration skills that it is too much to read. (Camilla)

It was suggested to include other modalities, such as videos, sounds, pictures and increase the interactivity in the program. The presentation of the treatment content was described as feeling a bit sterile, and proposed for the content to be more “attractive” and “cozier” in presentation (e.g., gamification) in order to make the content more accessible.

The usability of the guided iCBT program was also viewed as problematic for the therapists when delivering treatment. The webpage was by the majority of the therapists experienced as inconvenient to navigate, and required extensive clicking from page to page. The design of the pages was reported to be unintuitive, and therapists reported using time searching for information:

it is too difficult to find what I am looking for… and yeah, we use a lot of time going back and forth and wondering where things are and how to find them… it could be a lot more user-friendly…. (Monika)

The therapists requested more information on each page, and easier access to the most relevant information on the front page. Several therapists felt that usability challenges, to a degree, were compensated by the gain of more training and experience with using the webpage. However, most remembered their first impression of the page as being overwhelming.

It was overwhelming (…) and I wonder, if I felt it like that, how does it feel for the patients. (Sofie)

The user face of the program was designed a bit different from typical webpages, and this led to some usability problems for the therapists. The use of function keys, the limitation to have only one tab open at the same time, lack of undo buttons and long delays on actions felt unfamiliar to use, and led to errors that the therapists then had to contact superusers to fix. Furthermore, the therapists experienced poor interaction with other programs (e.g., the digital journaling system). Several therapists shared that they had to guide patients in use of the web-page, and that they missed a possibility to view the program from the patient-perspective.

I remember that I messed it up and started (the program for a patient) two times, because I could not immediately see that they… there is some delay when you have appointed the patient, and you have to wait until the program has given the patient access. And then I started two programs (for the same patient), as I got stressed when nothing happened, and assumed that I had done something wrong. (Anne)

Another interesting experience reported regarding delivering treatment using the guided iCBT program was that the program provided some emotional distance to the patients:

There was some more distance to it… you as a therapist could experience the same feelings, but in a… slightly milder form. So, it might be that you could think more clearly as a therapist. When you have more of an outside perspective and not are in the middle of it and get a little infected by… the feelings of hopelessness and helplessness… and everything the depression brings with it. (Maia)

The indirect manner and informal manner of how the program was designed also provided the opportunity to reflect and evaluate on messages before they were sent to the patients, which again could increase the possibility for the therapist to provide a more outside perspective. The distance might also make it easier for the patients to be more open with their therapists. One therapist revealed that some of the patients had written about quite personal topics that she believed she would never get divulged during face-to-face therapy sessions.

## 4. Discussion

Three themes were created to illuminate the therapist’s experiences providing guided internet-delivered therapy for patients with depression: (1) For the right person, at the right time; which covered the challenges therapists faced when appointing patients to the program. (2) It is not like chatting on Facebook; and the experienced demands that must be considered by clinics, therapists and patients, and the importance of contact between therapists and patients during treatment. (3) It is like a railroad, but without the switches, covers the experiences the therapists had using the program to deliver treatment to the patients.

### 4.1. Theme 1: for the right person, at the right time

It was quite clear from our data that the therapists perceived it to be challenging to predict which patients would benefit from the guided iCBT program, and this led to difficulties when appointing patients. Overall the therapists seemed to believe that the program was a good treatment option, but the challenge was in selecting it to *the right person, at the right time*. The recognition that internet-delivered treatment might not fit every patient is also discussed by therapists interviewed by Bengtsson et al. (2015). In Folker et al. (2018) it was discussed how the lack of flexibility in the programs makes it difficult to tailor the treatment to the patient’s needs, which also gets consequences for the intake of patients to the program. They conclude that more flexible programs would make it possible to include more patients, as personal needs could to a higher degree be accommodated.

The therapists in our study discussed that some dropout from the treatment should be expected. This is in line with findings from previous studies where dropout rates on CBT were investigated (Swift and Greenberg, 2012; Fernandez et al., 2015; Pentaraki, 2018; Schmidt et al., 2019). Dropout from the treatment should be anticipated, but it should also be emphasized that this guided iCBT program has demonstrated to be effective, also for patients who did not complete all modules (Jakobsen et al., 2017; Nordgreen et al., 2019). The demotivating feelings the therapists reported when patients dropped out of the iCBT might need to be addressed in training and introduction to the program. Dropouts from treatment should be discussed in light of previous findings that showed that dropout rates from CBT treatment and especially in the internet-delivered format could be high (Schmidt et al., 2019).

The high amount of responsibility placed on the patients to complete the treatment independently was also discussed as a potential risk. E.g. patients with a high symptom load making them in risk of not being able to take on this responsibility. Many reported that their patients struggled to find the time to prioritize the treatment in their busy everyday lives. The described need for motivation, independence, self-discipline and possibilities to prioritize the treatment were in line with the findings of Titzler et al. (2018) and Bengtsson et al. (2015) who found the patients interests, willingness and motivation as a key facilitator for the internet-delivered treatment. The problem that depressive symptoms in many cases affects these key factors were prominent in our data. For a patient group where symptoms could include loss of energy, diminished ability to think or concentrate and indecisiveness (DSM-5™, 2013) the intervention must be customized to meet these patients needs. Symptoms and other factors surrounding the patients should be considered when patient and therapist evaluate if guided iCBT treatment is the right treatment option. The timing of the treatment will also be crucial, to start treatment when the patient have a lot of other obligations or high burden of symptoms might be unfruitful.

### 4.2. Theme 2: it is not like chatting on Facebook

During the interviews with the therapists, it became evident that the treatment was not to be viewed as an easy option, and it was associated with high demands on clinics, therapists and patients. Unlike chatting on Facebook, the therapists described both their efforts and the patients’ efforts as quite extensive in order to provide or receive the effective treatment online.

#### 4.2.1. Sub-theme 2A: demands on the clinic and therapists

To have time devoted to the iCBT was important for the therapists. They emphasized the need for the leaders of clinics to prioritize and not underestimate the program and keep the therapists iCBT-time free of other tasks. The necessity for this was also found by Doukani et al. (2022), who described it as important that therapists, who provided internet-delivered therapy, did not experience time pressure. Time pressure could negatively impact the treatment and the therapeutic alliance between therapist and patient (Doukani et al., 2022). It appeared from our data that the iCBT-program needs to be better integrated into the appointments and scheduling systems at the clinics to reduce time pressure on the therapists. There seemed to be lacking a functioning system to keep allotted iCBT-time free of other appointments and tasks, and that the therapists were forced to find different strategies to cope with this challenge.

Similar to findings in other studies (Folker et al., 2018; Meisel et al., 2018; Titzler et al., 2018; Kerst et al., 2020; Smith and Gillon, 2021; Wood et al., 2021; Doukani et al., 2022; Vis et al., 2022), the therapists in this study expressed the need for sufficient training and supervision to provide good treatment. This might not be surprising, but as it is frequently reported it appears to be a challenge that should be considered and addressed when internet-delivered treatment is implemented. The clinics should have a plan for relevant and sufficient training, and secure that therapists have access to ongoing support (Meisel et al., 2018; Smith and Gillon, 2021; Wood et al., 2021; Vis et al., 2022).

The need for a supportive and accessible collegial community was reported as important for the therapists in the study, and was in line with the findings and recommendations from previous research (Meisel et al., 2018; Mol et al., 2020; Wood et al., 2021; Vis et al., 2022). Clinics aiming to offer internet-delivered treatment should ensure that several therapists could work together and support each other. This was described as important for good treatment delivery, and also as a positive wellbeing aspect for therapists.

The therapists described that the guided iCBT provided some variation in their workdays, which they enjoyed. Titzler et al. (2018) reported similar findings and described that varied activities positively compensated for the more demanding face-to-face sessions. The therapists in our study, even though they enjoyed providing iCBT, expressed that they would not have wanted to work fulltime with it. The variation was described as a key factor for job satisfaction. Clinics planning to implement therapist guided internet-delivered treatment should aim to combine it with face-to-face sessions and not demand that the therapists work solely in the internet-delivered format.

#### 4.2.2. Sub-theme 2B: demands on the patients

Wood et al. (2021) described that patients felt more autonomy when the treatment was delivered in an online format compared to traditional therapy, similar experiences were reported by therapists in our study. The iCBT appeared to give the patients more freedom and flexibility, but this also demanded that the patients take responsibility for their own treatment. Challenges and concerns related to this were expressed by the therapists, e.g., that the patients could procrastinate and avoid the treatment altogether. Several therapists described this as even more pressing in this format compared to face-to-face therapy. It might be that in face-to-face therapy it would be less apparent if the patients avoid or procrastinate tasks and assignments, as long as they continue to show up to their therapy appointments. But in the online format without face-to-face sessions, lack of engagement could be very noticeable. The patients’ motivation was therefore emphasized by the therapists as especially important, this was also reported by Titzler et al. (2018).

It appeared that when the patients were given more control over and responsibility for the treatment, some therapists found it uncomfortable and they expressed a feeling of risk (e.g., related to suicidality). To accept the boundaries of the treatment format, and deal with the feeling of less control is discussed in previous research (Smith and Gillon, 2021; Wood et al., 2021), and is something that should be considered both when appointing (Theme 1) and treating patients as well as during the training of therapists.

To give the patients more freedom and flexibility and by placing the majority of responsibility on them, it appeared to produce some positive effects in the patients. Therapists described it as possible for more patients with depression to get access to treatment, e.g., especially those patients who find it challenging to have regular appointments at the therapist’s office. Access to treatment is a challenge worldwide (Kessler et al., 2003; Wang et al., 2007; Kazdin, 2017; Thornicroft et al., 2017), and providing therapy online could be a way to increase access.

The high demands placed on patients with online therapy was described by therapists as empowering to those patients who were better suited to treat themselves as well as promising in preventing relapses. These findings were also supported by previous published research (Titzler et al., 2018). To have the feeling of being in control over your own health was described as pertinent for keeping patients engaged in their digital mental health interventions (Borghouts et al., 2021). Thus, the format of guided iCBT seemed to support this. But, as reported by the therapists, this also calls attention to patients’ autonomy in that they actively choose this treatment, they are committed to and want to complete it.

As the focus in this study was to investigate the therapists’ experiences with the treatment and only therapists were interviewed, the possibilities to draw conclusions about the patients’ experiences is limited. Other demands could be experienced by the patients.

#### 4.2.3. Sub-theme 2C: the need for contact between patients and therapists

The interviewed therapists described the contact between them and the patients as a valuable part of the guided iCBT. The programs secure email-system functioned as a channel for encouragement, motivation and help. Contact over mail, and in some cases phone calls and/or face-to-face sessions were described as important to build a relationship with the patients. As found in previous research, building relationships and a therapeutic alliance is possible in digital interventions, but might be more challenging compared to traditional therapy (Bengtsson et al., 2015; Meisel et al., 2018; Titzler et al., 2018; Kotera et al., 2021; Doukani et al., 2022). Similar to reports by therapists in Khan et al. (2022) and Wood et al. (2021) the therapists in our study generally reported building good relationships with their patients in iCBT, but many described that certain meetings with the patient (e.g., conducting the intake interview) were beneficial. This could indicate that clinics should facilitate for meetings between patients and therapists, and have the e-therapists conduct intake interviews as this could be an important start of the therapeutic relationship.

An interesting finding was the emphasis the therapists put on the messages they wrote to the patients. Similar to findings by Khan et al. (2022) some therapists in our study expressed that the text-based format of the iCBT had some advantages over face-to-face treatment in that the feedback to patients could be reread, discussed with colleagues, edited and reflected upon before communicating back to the patient. The program provided pre-written messages, which the therapists reported as seldomly used. This might indicate that the mail function was viewed as a possibility for the therapist to have some influence on, and tailor the intervention for the patients, which is a frequently described challenge and potential barrier for internet-delivered treatment (Folker et al., 2018; Meisel et al., 2018; Titzler et al., 2018; Borghouts et al., 2021; Kotera et al., 2021; Doukani et al., 2022). This might explain why therapists reported spending too much time on formulating messages, and instead offered meetings or phone conversations with patients, when deemed as necessary. In addition, this might point to the needs certain specific patients require. Some therapists expressed that certain outpatient clinic patients were at the borderline of being too ill to be selected for iCBT, but by compensating with frequent and comprehensive contact might be a possible solution to support these patients.

### 4.3. Theme 3: “it is like a railroad, but without the switches”

The third theme we created embraced how the therapists experienced to use the iCBT program to deliver treatment content to their patients.

A common experienced or anticipated barrier to internet-delivered treatment was technical difficulties (Titzler et al., 2018; Mol et al., 2020; Borghouts et al., 2021). Similar to findings by Khan et al. (2022) the therapists in our study did not experience that technical difficulties affected the building of relationships with their patients or the delivery of the treatment. This might indicate that the therapists had access to necessary resources, reliable software, good technical solutions and technical support, which were important prerequisites for providing internet-delivered therapy (Vis et al., 2022). The therapists expressed that they experienced good data protection and security in the iCBT program, which was supported by research that had investigated data protection and security for this specific iCBT program (Yogarajah et al., 2020).

Some therapists expressed that the pre-set structure of the iCBT program had some advantages, as the format secured more control over the treatment the patients received. This was in line with reports from therapists in Mol et al. (2020), who described more adherence to treatment protocol and less therapist drift. But the pre-set structure was generally viewed as problematic as it made the program inflexible and unadaptable to the patient’s specific needs. Lack of possibilities to tailor the treatment to each patient was frequently reported as a potential problem in internet-delivered treatment programs (Folker et al., 2018; Titzler et al., 2018; Mol et al., 2020; Borghouts et al., 2021; Doukani et al., 2022). To make changes based on the patients’ needs are common in traditional therapy, and the lack of possibilities to do this made one therapist compare the program to a railroad without switches, a train that cannot change direction once it has started moving. The therapists expressed that they used different methods to compensate for this lack of flexibility, similar to findings presented by Mol et al. (2020). Therapists also expressed that many of the patients were too ill, and the inflexibility made the program feel like it was a “one size fits all” solution, and this is where the guided iCBT as a stand-alone intervention in many cases fell short. These topics could be related to other themes: only the right patients should be appointed to the treatment and that the program puts high demands on clinics, therapists and patients and cannot be taken lightly.

Similar to our findings, to base the delivery of treatment content on presenting text to the patients is commonly reported to be associated with some challenges (Sinha Deb et al., 2018; Gullickson et al., 2019; Mol et al., 2020; Borghouts et al., 2021). To include more modalities (e.g., videos, sounds, interactive tasks) and gamification of the treatment content has been discussed in previous studies (Sardi et al., 2017; Andersson et al., 2019; Six et al., 2021) as well as proposed by the therapists in this study. Six et al. (2021) found that gamification in mental health apps directed at patients with depression did not influence the effectiveness (adherence and reduction of symptoms) of the intervention. While therapists in our study frequently cited there was too much text with the current iCBT program, an aim for future development should be to design online interventions that are meaningful, attractive and useful to patients (Yardley et al., 2015).

Usability was previously reported as a challenge in digital mental health interventions (Yogarajah et al., 2020), and specifically for patients in the eCoping program (Nordgreen et al., 2019). With the focus in this study being on the therapists’ experiences, usability was discussed based on how the program was viewed from the therapists’ perspectives when they provided therapy to their patients. Generally, the therapists reported that the usability was satisfactory, but that there was room for improvement. The therapists expressed that there was too much clicking, unintuitive navigation on the pages (e.g., use of the function keys), lack of an undo option, and delays in the system that made the program inconvenient to use. The program also interacted poorly with other programs used (e.g., the journaling system). Normal navigation on computers and the internet generally includes immediate feedback from a user’s actions (Nah, 2004; Basalla et al., 2021), and an interface with longer delays could feel unfamiliar to use, and thus, errors should be expected, especially with unexperienced users (Basalla et al., 2021). In line with previous research (Nah, 2004; Kim et al., 2017) this illuminated the need for more immediate feedback from actions at the iCBT programs webpages. The possibility of having more pages or tabs open at the same time was also something the therapists missed. An easy and accessible undo-option should also be added, as well as clickable buttons to replace the use of function keys. It might be considered a risk that the therapists get access to too many of the functions in the program, and that some functions should only be appointed to administrators only. But as it is experienced now, it appears that this limited access to functions has also led to problems. It is also possible that the amount of errors that needs to be corrected would decrease if the usability of the program was better. Lastly the possibility to view the intervention from the patient’s perspective would make guidance and support for the patients easier. This is important for the delivery of treatment content since those patients who experience difficulties with navigating the program-pages, will most likely have trouble accessing all needed treatment content. In line with findings from Wood et al. (2021) the therapists should get sufficient and specific training in use of the program-pages especially as it has been perceived by many therapists as unintuitive.

One interesting and quite contradicting finding was how the therapists reported that the patients felt close, at the same time as they reported more emotional distance compared to traditional therapy. Similar to findings reported by Wood et al. (2021) some therapeutic relationships were developed quickly, with therapists finding patients were more rapid in their sharing of their personal thoughts, feelings and experiences. Similar findings were reported by Bengtsson et al. (2015). This might be attributed to the informal, and easily accessible mail-system, which also provided some distance. As described by Bengtsson et al. (2015), the textual format might have felt less confronting than a physical meeting. The indirect contact in writing might have made sharing easier and facilitated more openness. The distance seemed to make the therapists less affected by the patient’s depression, and able to think clearer when providing feedback. Bengtsson et al. (2015) reported similar experiences among Swedish therapists and speculated that internet-delivered therapy could prevent exhaustion in the therapists. These findings should be viewed in light of Khan et al. (2022) who found that therapists were able to stay present and attuned to patients’ emotions even when treatment was delivered through an online format. To provide a treatment program where therapists could stay cognizant with the patient’s emotions as well as have an emotional distance that makes them less affected by the patient’s symptoms seems to be a promising feature of the internet-delivered treatment.

### 4.4. Limitations

The process of recruiting and carrying out the interviews was extensive. The first interviews were conducted in December 2020, and the last interview in March 2022. Many of the therapists interviewed later in the study had limited experience with the guided iCBT program (1–5 patients), but most had worked as therapists during the COVID-19 pandemic and had some experience providing remote therapy. Most of the therapists had also used other versions of the iCBT directed at patients with other disorders (e.g., anxiety).

A more comprehensive and in-depth analysis might have been provided if more therapists were recruited and shared their experiences with the program. However, the number of participants in this study was in line with guidelines in terms of participants needed to conduct a reflexive TA (Braun and Clarke, 2013) and given the breadth and depth of data each participant provided, the enrolment number seemed suitable for the study.

### 4.5. Future studies

Future studies including focus-group interviews where therapists could discuss their experiences with each other, could yield interesting results. This was not possible in this study due to the pandemic. Utilizing a focus-group interview structure, the therapists would be able to discuss the program with each other, which could yield topics not considered in this study.

Future studies should also aim to gather information about the patients’ perspectives with using the program to provide a more comprehensive analysis of how they have experienced it. The patients’ perspectives are important and should be included when designing, implementing and evaluating digital interventions (Yardley et al., 2015), which would be the ultimate aim of this research.

## 5. Conclusion

By the use of reflexive TA, we aimed to explore therapists’ experiences providing internet-delivered therapy for depression. With open-ended questions in a semi-structured interview we aimed to gather data that would provide an in-depth account of the challenges experienced by the 12 therapists who used a guided iCBT program in the treatment of their patients. The aim of this study was to gather insight into what therapists experienced from providing guided iCBT to patients with mild and moderate depressive disorder.

Three themes that were created, covered what we considered important aspects of the therapists’ experiences: (1) For the right person, at the right time; (2) It is not like chatting on Facebook; (3) It is like a railroad, but without the switches. Generally, the therapists were positive towards the program and found it helpful for their patients. The problem of selecting the right patients to secure treatment success was prominent. The online-format implied that compared to traditional face-to-face therapy, the additional demands placed on clinics, therapists and patients with online therapy needed to be addressed. The online-format also seemed to increase the value of the contact between the patients and therapists, and the therapists used time and effort to provide meaningful, supportive and challenging messages to their patients. The therapists expressed some concerns with the usability of the program, and reported need for more flexibility and possibilities to tailor the treatment for each patient.

The challenges and benefits reported in this study are generally in line with previous research. The notion that the format might not fit every patient is important to acknowledge and in line with findings by Bengtsson et al. (2015). That the treatment places high demands on patients and therapists and accommodations might be necessary is also supported by previous research (Meisel et al., 2018; Titzler et al., 2018; Smith and Gillon, 2021; Wood et al., 2021; Doukani et al., 2022; Vis et al., 2022), as well as the experience that a therapeutic relationship could be built even in this format (Wood et al., 2021; Khan et al., 2022). Challenges, but also benefits from delivering treatment content in this format was also in line with previous reports of a need for tailoring to patient’s needs (Folker et al., 2018; Titzler et al., 2018; Mol et al., 2020; Borghouts et al., 2021; Doukani et al., 2022), usability problems (Nordgreen et al., 2019) and that the format could contribute to some emotional distance for the therapists (Bengtsson et al., 2015).

## Data availability statement

The original contributions presented in the study are included in the article/Supplementary material, further inquiries can be directed to the corresponding author.

## Ethics statement

The studies involving human participants were reviewed and approved by the Norwegian regional committees for medical and health research ethics. The participants provided their written informed consent to participate in this study.

## Author contributions

LB, TN, and AN-H contributed to conceptualization and design of the study. LB conducted data collection and analysis with guidance and supervision from TN and AN-H. LB wrote the first draft of the manuscript. All authors contributed to the article and approved the submitted version.

## Conflict of interest

The authors declare that the research was conducted in the absence of any commercial or financial relationships that could be construed as a potential conflict of interest.

## Publisher’s note

All claims expressed in this article are solely those of the authors and do not necessarily represent those of their affiliated organizations, or those of the publisher, the editors and the reviewers. Any product that may be evaluated in this article, or claim that may be made by its manufacturer, is not guaranteed or endorsed by the publisher.
