# Corrigendum: Therapists' experiences with providing guided internet-delivered cognitive behavioral therapy for patients with mild and moderate depression: a thematic analysis

**DOI:** 10.3389/fpsyg.2023.1274464

**Published:** 2023-12-19

**Authors:** Line Børtveit, Tine Nordgreen, Anders Nordahl-Hansen

**Affiliations:** ^1^Faculty of Health, Welfare and Organisation, Østfold University College, Halden, Norway; ^2^Department of Behavioral Sciences, Faculty of Health Sciences, Oslo Metropolitan University, Oslo, Norway; ^3^Division of Psychiatry, Haukeland University Hospital, Bergen, Norway; ^4^Department of Global Public Health and Primary Care, University of Bergen, Bergen, Norway; ^5^Department of Education, ICT, and Learning, Østfold University College, Halden, Norway

**Keywords:** internet-delivered therapy, therapists' experiences, providing online therapy, iCBT, internet-delivered therapy for depression, thematic analysis

In the published article, there was an error in the article title. Instead of “Therapists' experiences with providing guided internet-delivered cognitive behavioral therapy for patients with mild to moderate depression: a thematic analysis,” it should be “Therapists' experiences with providing guided internet-delivered cognitive behavioral therapy for patients with mild and moderate depression: a thematic analysis.”

Additionally, in the published article, there was an error in the text. In several places, the word “to” was used instead of the word “and” when describing that the therapists had experience providing therapy for patients with both mild and moderate depression.

A correction has been made to the **Abstract**, sub-section “*Introduction*.” This sub-section previously stated:

“**Introduction:** Guided internet-delivered therapy has shown promising results for patients with mild to moderate depressive disorder, but several challenges with the format have been reported. The aim of this qualitative study was to investigate therapists' experiences providing guided internet-delivered cognitive behavioral therapy for patients with mild to moderate depression.”

The corrected sentence sub-section below.

“**Introduction:** Guided internet-delivered therapy has shown promising results for patients with mild and moderate depressive disorder, but several challenges with the format have been reported. The aim of this qualitative study was to investigate therapists' experiences providing guided internet-delivered cognitive behavioral therapy for patients with mild and moderate depression.”

A correction has also been made to **1. Introduction**, “*1.1. Internet-delivered therapy*,” paragraph 1. The incorrect paragraph previously stated:

“Meta-studies have shown that therapist guided internet-delivered interventions are effective in treatment for mild to moderate depressive disorders, and few patients (5%-10%) experience deterioration or negative effects (Cuijpers et al., 2015; Rozental et al., 2015; Ebert et al., 2016; Karyotaki et al., 2018; Etzelmueller et al., 2020; Chan et al., 2022; Hedman-Lagerlöf et al., 2023).”

The corrected paragraph appears below.

“Meta-studies have shown that therapist guided internet-delivered interventions are effective in treatment for mild and moderate depressive disorders, and few patients (5%-10%) experience deterioration or negative effects (Cuijpers et al., 2015; Rozental et al., 2015; Ebert et al., 2016; Karyotaki et al., 2018; Etzelmueller et al., 2020; Chan et al., 2022; Hedman-Lagerlöf et al., 2023).”

A correction has been made to **1. Introduction**, “*1.3. Purpose and aim*,” paragraph 2. This sentence previously stated:

“The purpose of this study is to investigate therapists' experiences providing guided iCBT to patients with mild to moderate depressive disorder.”

The corrected paragraph appears below.

“The purpose of this study is to investigate therapists' experiences providing guided iCBT to patients with mild and moderate depressive disorder.”

A correction has been made to **2. Materials and methods**, “*2.1. The eCoping program*.” This sentence previously stated:

“The eCoping program is a therapist guided iCBT intervention for patients with mild to moderate depression.”

The corrected paragraph appears below.

“The eCoping program is a *therapist guided* iCBT intervention for patients with mild and moderate depression. The eCoping program is based on an intervention designed by Andersson et al. (2005) and adapted to Norwegian users. The program is module based and consists of eight modules, all modules and a short description of their content is presented in Table 1. The program is completed over approximately 14 weeks. Asynchrony contact with feedback and guidance is provided in an integrated secure email-system after completion of modules, but more contact (e.g., over phone) is provided as needed. The program has effectively been used in outpatient clinics in Norway (Jakobsen et al., 2017; Nordgreen et al., 2019).”

A correction has been made to **2. Materials and methods**, “*2.4. Researcher positioning*.” This sentence previously stated:

“She has no former experience with providing therapy or working with patients who are diagnosed with mild to moderate depression.”

The corrected paragraph appears below.

“Given the researchers' active roles in reflexive TA, the context or positioning of the researchers should be described (Braun and Clarke, 2006, 2023). LB is a researcher working on a project focusing on internet-delivered treatment for depression. She has no former experience with providing therapy or working with patients who are diagnosed with depression. When conducting the interviews with the therapists, she informed about her “outsider” perspective. TN has been working with the development, evaluation and implementation of guided iCBT for the last 15 years. AN-H is a researcher in the transitional fields of special education, psychology and psychiatry, and have clinical experience working in the psychiatric field for 12 years.”

Lastly, a correction has been made to **5. Conclusion**, paragraph 1. This sentence previously stated:

“The aim of this study was to gather insight into what therapists experienced from providing guided iCBT to patients with mild to moderate depressive disorder.”

The corrected paragraph appears below.

“By the use of reflexive TA, we aimed to explore therapists' experiences providing internet-delivered therapy for depression. With open-ended questions in a semi-structured interview we aimed to gather data that would provide an in-depth account of the challenges experienced by the 12 therapists who used a guided iCBT program in the treatment of their patients. The aim of this study was to gather insight into what therapists experienced from providing guided iCBT to patients with mild and moderate depressive disorder.”

The authors apologize for these errors and state that they do not change the scientific conclusions of the article in any way. The original article has been updated.
