# Supplementary material for: Therapists’ experiences with providing guided internet-delivered cognitive behavioral therapy for patients with mild and moderate depression: a thematic analysis
Source: Front Psychol. 2023 Jul 14;14:1236895. doi: 10.3389/fpsyg.2023.1236895 (PMC10380928; doi:10.3389/fpsyg.2023.1236895)
Supplement: Supplementary file 1 [file Data_Sheet_1.docx]

Supplementary Material

# Appendix A: Interview guide (Translated from Norwegian)

## Introduction

Inform the participant about the aim of the interview:

“The aim of the interview is to gather information from those who have used the treatment program: about things that works, and things that should be improved. The feedback from you, and other therapists will be used for further development of the treatment program”.

Inform about **how** the interview will be conducted:
“I am going to ask quite open-ended question, some of them will be quite general. You should not be afraid that you answer a question that might come later in the interview. Answer with what you find important, and whatever that comes to mind first. It is not a problem if you during the interview thinks of something you would add to an earlier question”.

Inform about the use of **audio-recording** of the interview, and why this is necessary to secure good data collection. Inform about how the audio-files and other information about the participant will be stored.

Inform the participant about possibilities to take **breaks** whenever they need it, and that the interview is estimated to take about one hour to complete.

Inform about how the information from the interviews also will be used as a part of a phd-thesis, and that the goal is that the results should be **published** in a scientific journal. The participants would be anonymized in the article, and it would not be possible to see who participated in the study.

## Interview questions

### Intervention:

Generally, /overall (the larger picture): how did you experience this treatment program?

How was it to provide treatment in this way?

What do you think influenced if the treatment worked or not worked for your patients?

What do you think about the content of the treatment?

How important was if for you to have contact with your patients using the webpage?

Was there something in the treatment program that did not work well for you as a therapist?

### Facilitation:

Were arrangements made for this way of working at your workplace?

Did you receive any training in use of the program?

Did you receive guidance/supervision, how much guidance did/do you get?

Were you several colleagues that worked with this at the same time, or were you alone with your patients? Did you have a commonality where you could discuss challenges and things that happened during therapy?

How much time did you use per patient? For instance, compared to traditional therapy?

Was it difficult to combine this treatment program with your work days? For instance, was it challenging to find time to follow up on modules and tasks? Challenging to log on to the web-page, register patient activity etc.?

### Usability:

#### The treatment program:

How was it to be a therapist and use this treatment program?

How did you experience to work with the different modules?

Is there anything about the modules that you think should be changed, or does they function well as they are?

What do you think influenced if your patients did, or did not do their tasks?

#### The web-pages

How did you experience to use the webpage?

Did the webpage work? (or did you for example experience technical issues along the way?)

How was it to log on?

How was it to navigate the webpage?

Try to remember your first encounters with the webpage, how did you experience it? Did you find what you needed immediately, or did you have to search for it? What was your first impressions?

Do you have any ideas on how to make the webpage better?

## Acceptability of the treatment:

What do you think about this way to provide treatment?

Do you think this treatment is full-fledged or is there something you are missing? If this is the case, what do you miss?

If treatment for patients with depressions in the future should be more like this treatment program, do you have any input about things that should be changed for it to be better to be a therapist?

## Satisfaction:

Are you satisfied with how you experience this treatment program?

Would you recommend this kind of treatment to friends or acquaintances with depression?

## **Closing questions**:

Is there something you would like to add, now that we have reached the end of the interview?

How was it to be interviewed about the treatment program?

# Concluding information:

Contact information to the first and second author, in case of questions or comments after the interview.

Information about the possibility to withdraw from the study, and get handed out all gathered information and data about the participant.

# Questions after the interview:

How many patients (approximately) do you estimate to have worked with using the eCoping program for depression.

What is your education/background?

Where should we send the “thank-you” gift card.
