# Supplementary material for: Therapists’ experiences with providing guided internet-delivered cognitive behavioral therapy for patients with mild and moderate depression: a thematic analysis
Source: Front Psychol. 2023 Jul 14;14:1236895. doi: 10.3389/fpsyg.2023.1236895 (PMC10380928; doi:10.3389/fpsyg.2023.1236895)
Supplement: Supplementary file 2 [file Data_Sheet_2.docx]

Supplementary Material

# Appendix B: Definitions of themes and subthemes

## Theme 1: “For the right person, at the right time”

The first theme is about experiences the therapists have with inclusion to the treatment, and how they reported this as a challenging task.

This theme includes:
Experiences the therapists have with inclusion to the program, which patients fits, and which patients should be offered other treatment options.
The difficulties therapists report with predicting treatment success.
Experiences with patients that succeed in the program, and characteristics of these patients.

Theme 1 does not include:
How the therapists adapts or tailor the treatment to better fit the patients (linked to theme 2 and 3).
Discussions about usability and how the content is conveyed to the patients (theme 3).
How the therapists work with the patients in the program: contact etc. (theme 2 and 3).
The demands the program puts on the patients (theme 2).

## **Theme 2: “It is not like chatting on facebook”**

This theme is about how the therapists reported that the program should not be viewed as a simple treatment option, but as a treatment that put high demands on therapists/clinics and patients, and contact is an important part of the treatment.

### Subtheme 2a) Demands on the clinics and therapists.

This subtheme includes:
Reports from the therapists about the time and effort they put into the treatment, and their expressed need for this to be accommodated by the clinics: e.g. keep the allotted time free of other tasks.
The expressed need for the clinics to provide sufficient training, guidance and facilitate a collegial community: e.g. have a team, shared offices etc.

### Subtheme 2b) Demands on the patients.

This subtheme includes:

Reports from the therapists about the high amount of effort the patients must put into this treatment.
The patients are described as being their own therapists: and the sub-theme includes benefits and challenges regarding this, especially the problem with motivation, initiative, concentration and attention during depression.

### **Subtheme 2c) The need for contact**

This subtheme includes:
Reports from the therapists about the value of having contact with the patients during treatment.
How balance between providing support, guidance, help, motivation, encouragement- and nagging.

The use of pre-written messages, and the emphasis on what they convey to patients.
The value of building a relation. The feeling of being close at the same time as the program provides some emotional distance.

Theme 2 does not include:

Experienced demands regarding inclusion and exclusion of patients to the program (linked to theme 1)

Discussions about the treatment content and demands therapists have to the content (e.g. what the modules should cover, the treatment approach).
Demands the therapists have about the program-pages regarding usability and conveying of treatment content: e.g. the amount of text, the wordings etc (theme 3).

## Theme 3 “It is like a railroad, but without the switches”

This theme is about delivering treatment using the program.

Theme 3 includes:
Reports about how content is conveyed: e.g. to much text, lack of interaction, gamification, how tasks are presented.
How the therapists experience the usability of the program.
Reports of lack of flexibility and possibilities to tailor the treatment to each patient, and how the therapists dealt with this.

Theme 3 does not include:
Considerations about the content of the treatment.
The time, effort and accommodations necessary for the therapists to provide this therapy (linked to theme 2).
Characteristics of the patients that would benefit from having treatment content delivered in this manner (linked to theme 1).
